# Laser Actuated Microgripper Using Optimized Chevron-Shaped Actuator

**DOI:** 10.3390/mi12121487

**Published:** 2021-11-30

**Authors:** Belal Ahmad, Hugo Chambon, Pierre Tissier, Aude Bolopion

**Affiliations:** FEMTO-ST Institute, Université Bourgogne Franche-Comté, CNRS, 25000 Besançon, France; chambon.hugo@gmail.com (H.C.); pierre.tissier@icm-institute.org (P.T.); aude.bolopion@femto-st.fr (A.B.)

**Keywords:** microgripper, micromanipulation, optothermal actuation

## Abstract

In this paper, we propose a laser actuated microgripper that can be activated remotely for micromanipulation applications. The gripper is based on an optothermally actuated polymeric chevron-shaped structure coated with optimized metallic layers to enhance its optical absorbance. Gold is used as a metallic layer due to its good absorption of visible light. The thermal deformation of the chevron-shaped actuator with metallic layers is first modeled to identify the parameters affecting its behavior. Then, an optimal thickness of the metallic layers that allows the largest possible deformation is obtained and compared with simulation results. Next, microgrippers are fabricated using conventional photolithography and metal deposition techniques for further characterization. The experiments show that the microgripper can realize an opening of 40 µm, a response time of 60 ms, and a generated force in the order of hundreds of µN. Finally, a pick-and-place experiment of 120 µm microbeads is conducted to confirm the performance of the microgripper. The remote actuation and the simple fabrication and actuation of the proposed microgripper makes it a highly promising candidate to be utilized as a mobile microrobot for lab-on-chip applications.

## 1. Introduction

The manipulation of micro-sized objects has drawn more attention in recent years to advance highly demanding domains such as microassembly and biomedicine [1,2,3]. Specifically, microgrippers are one of the widely used devices to handle microobjects [4,5,6]. At this scale, different actuation approaches, such as electric [7,8], thermal [9], magnetic [10] and optical [11], are used to control the motion of the microgripper, where piezoelectric and electrothermal actuation are most dominant in commercialized microgrippers. Since many microscale applications, especially biomedical applications, are conducted in closed environments, a remote actuation scheme is advantageous [12,13,14]. In fact, optothermal actuation, where light is utilized to generate heat at a specific location in an object, is one of the promising actuation approaches for microgrippers. Its remote and localized nature makes it a suitable candidate for mobile applications in closed environments [15]. Moreover, it allows simple integration of the microgripper in mobile microrobots, which are widely actuated by magnetic or acoustic fields, without affecting the actuation of the microrobot itself.

A number of works have utilized the optothermal response to develop microactuators and microgrippers. In general, optothermal microactuators and microgrippers are mainly implemented using smart material designs [16], or chevron-shaped actuators [17]. In smart material design, a photoresponsive material is mixed with a flexible material, e.g., polymers, to achieve a bending motion upon illumination [18]. These materials provide highly flexible motion that can be further controlled by patterning the mixed material through advanced techniques such as shape programming [19]. Nonetheless, this comes at the cost of increased fabrication complexity. On the other hand, the chevron-shaped actuators, which are commonly based on polymers and are fabricated using conventional photolithographically techniques, offer an effective actuator that can generate relatively large displacement without complex fabrication techniques, in contrast to smart materials. Although the flexibility and range of motion of chevron-shaped actuators is lower than their smart materials counterparts, they offer a comparatively high generated force, which is critical for micromanipulation [20]. For instance, Elbuken et al. [17] have developed a photothermally actuated microgripper fabricated with a single polymeric layer (SU-8) and based on a chevron-shaped actuator. The microgripper could be actuated remotely to realize an opening of 30 µm using a focused laser beam to heat up a connection spot between two beams in the chevron-shaped structure. However, a common problem when utilizing the photothermic behavior of polymeric actuators is their low optical absorbance [21], which drastically reduces the overall displacement of such actuators. Since chevron-shaped actuators can be fabricated with common microelectromechanical systems (MEMS) fabrication techniques, a viable workaround is to use metallic coating to enhance the optical absorbance. By using a metal with high optical absorbance, the overall deflection of the chevron-shaped actuator can be increased. Moreover, the high absorbance of optical energy drastically enhances the response speed of the actuator. Specifically, gold is a superior absorbent of visible light and has shown high potential in optothermal actuation [22,23]. For example, gold nanoparticles have been integrated in a tunable biopolymer material to facilitate its optothermal response [24]. Moreover, gold is a commonly available material in cleanrooms with a well know deposition processes. Still, the integration of gold in microactuators increases its fabrication complexity. In this paper, we propose a polymeric microgripper utilizing a gold metallic coated chevron-shaped actuator that can be actuated remotely with a laser. Our aim is to overcome the low optical absorbance limitation of polymers by introducing a gold metallic layer with optimal thickness that would not affect the flexibility of the polymer itself. The thickness of the metallic layer is optimized to realize the largest possible opening of the microgripper. By virtue of the metallic layer, the proposed microgripper can realize large displacements and a fast response compared to previously developed polymeric microgrippers. To our knowledge, this work is the first attempt to enhance the optothermal actuation of chevron-shaped microgrippers using metallic coating. The untethered nature of laser actuation makes it possible to integrate the microgripper in a mobile microrobot in future work.

## 2. Microgripper Design, Optimization, and Fabrication

In this section, the microgripper design and the base material choice are first introduced. Next, the metal-coated chevron-shaped actuator, which is the main component of the microgripper, is optimized to realize relatively large deflections. Finally, the fabrication process of the microgripper is shown.

### 2.1. Design of Microgripper

**Figure 1:**
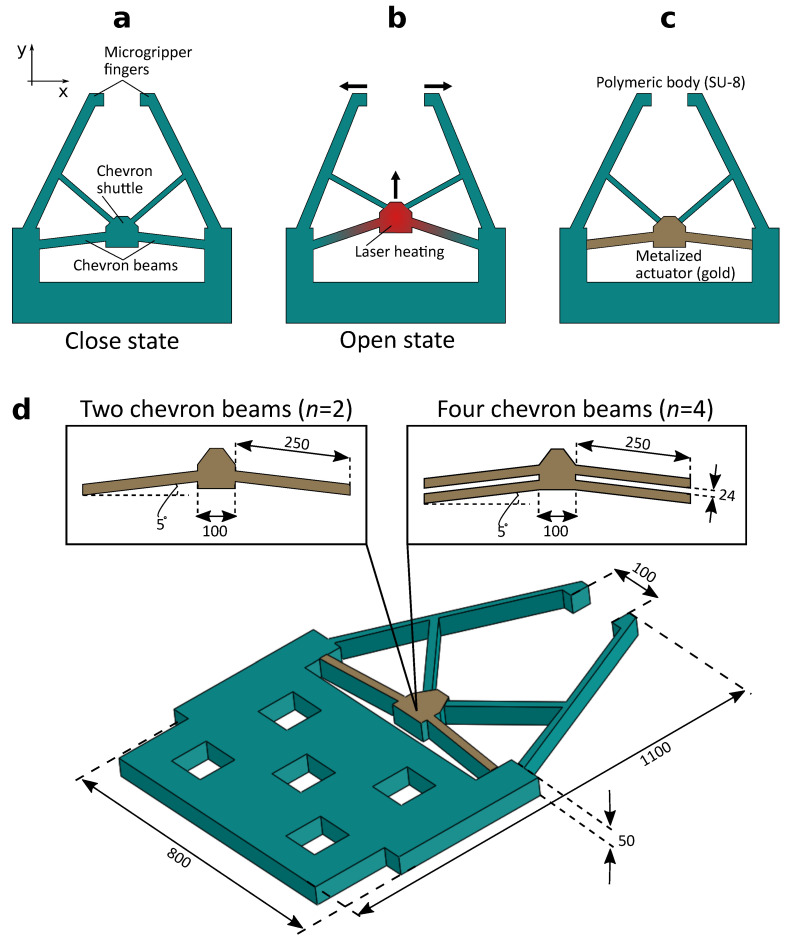
(**a**) Schematic of the proposed microgripper. (**b**) Laser heating of the chevron-shaped actuator results in opening the microgripper. (**c**) Material structure of the microgripper. (**d**) Dimensions of the microgripper. Two possible designs with two chevron beams (n=2) and four chevron beams (n=4) are shown. All dimensions are in µm.

The main component of the proposed microgripper is a chevron-shaped thermal actuator that produces translational displacement when heated. The two beams of the actuator, called “chevron beams”, are connected to the body of the microgripper from one end, acting as a fixed end, and to a common connection point, called “chevron shuttle”, acting as a free end, as shown in Figure 1a. It is worth noting that it is possible to add more pairs of chevron beams to the design as will be shown in the experimental section. Upon heating the chevron shuttle using a focused laser beam, the heat is conducted through the chevron beams causing them to expand, creating a translational displacement at the shuttle, as shown in Figure 1b. This displacement is utilized to push the fingers of the microgripper, which are normally in a closed state, further apart achieving an open state. Specifically, the normally closed state allows the microgripper to hold and move microobjects without being continuously heated by the laser beam, which is preferable to reduce the amount of heat dissipated in the surrounding environment.

The material structure of the microgripper is shown in Figure 1c. SU-8 resin was chosen as the body material of the microgripper. The use of SU-8 offers a number of advantages in microfabrication and thermal actuation. Since SU-8 is a very common material in MEMS manufacturing, its fabrication process is straightforward and it can be deposited in a thick layer (several ten microns). In addition, SU-8 is known to produce relatively large displacements in response to temperature change owing to its high thermal expansion coefficient [25]. On the other hand, the use of SU-8 poses a number of disadvantages especially in the case of optothermal actuation. Specifically, the thermal conduction coefficient of SU-8 is low, which is both advantageous and disadvantageous. In fact, when heating the chevron-shaped actuator with a laser, the low thermal conduction coefficient of SU-8 drastically reduces the amount of heat conducted from the heated actuator to the fingers of the microgripper. This is advantageous in biomedical applications to prevent damage to the manipulated biological entities. However, the disadvantage is the low temperature conduction from the heated shuttle to the beams of the chevron-shaped actuator, which reduces its overall displacement. Moreover, the transparent nature of SU-8 drastically reduces its optical absorbance; thus reducing its deformation when heated using a laser beam.

To compensate for the transparency of SU-8 and to enhance its optical energy absorption, a thin layer of a high optical absorbance metal is selectively deposited on the chevron-shaped actuator. The metallic layers are deposited on both sides of the actuator with equal thicknesses to reduce the bimaterial effect. In fact, the thickness of the metallic layer is a key parameter to achieve the largest possible deflection. On the one hand, an excessively thick layer would increase the stiffness of the chevron-shaped actuator reducing its deflection. On the other hand, an excessively thin layer would suffer from low optical absorbance. Therefore, the thickness of the metallic layer should be optimized for maximal possible deflection. The full design and dimension of the microgripper are shown in Figure 1d. The figure demonstrates the ability to vary the number of chevron beams through two examples of chevron actuators having two (n=2), or four (n=4) chevron beams.

### 2.2. Modeling and Optimization of Metal-Coated Chevron-Shaped Actuator

**Figure 2:**
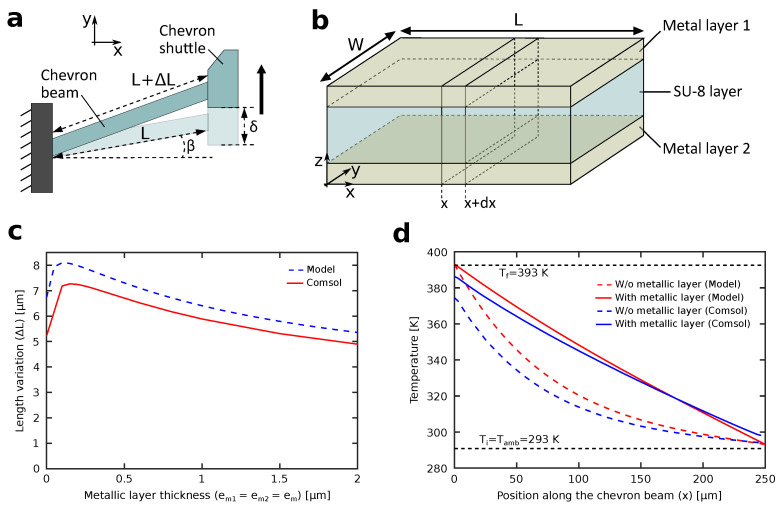
Modeling of the thermal deformation of a metal coated chevron-shaped actuator. (**a**) Schematic of the deflection of a half chevron-shaped actuator upon heating. (**b**) A sectional view of the chevron beam. (**c**) Effect of the thickness of the metallic layer on the length variation of the chevron beam. The blue dashed line and the red solid line show the model calculations and the simulation results, respectively. (**d**) Effect of adding metallic layers on the temperature gradient of the chevron actuator. The metallic layers thicknesses $e_{m1}=e_{m2}=e_{m}=200$ nm. The red solid line and the red dashed line show the model calculations for the cases with and without metallic layers, respectively, and the blue line and the blue dashed line show the simulation results for the cases with and without metallic layers, respectively.

In order to optimize the design of the chevron-shaped actuator, a model should be first derived. Because the actuator is symmetrical on the yz-plane, a model of only half of the actuator cut in the yz-plane is derived for simplicity. A schematic of the deflection of a half chevron-shaped actuator upon heating is shown in Figure 2a. The translational motion (δ) of the chevron shuttle can be estimated as a function of the variation in the length of the chevron beam (ΔL) as follows:

(1) $$\begin{array}{c} \delta =\sqrt{2L\Delta L+\Delta L^{2}+L^{2}\sin^{2}\left(\beta \right)}-L\sin\left(\beta \right) \end{array}$$

(2) $$\begin{array}{c} \Delta L=\int_{0}^{L}T\left(x\right)\alpha \;dx \end{array}$$

where *L* is the length of the chevron beam, β is the angle of the chevron beam with respect to the *x*-axis, T(x) is the temperature of the cross section of the chevron beam located at *x* coordinate, and α is the thermal expansion coefficient. From the model, it can be confirmed that the deflection of the chevron shuttle (δ) increases with decreasing angle of the chevron beam (β). The temperature conduction along the chevron beam can be modeled similar to the heat transfer from a fin by assuming that the temperature is constant over a small length dx as shown in Figure 2b. The temperature conduction should take into account the different thermal conductivity of the the SU-8 body and the two metallic layers. The two metallic layers have equal thicknesses ($e_{m1}=e_{m2}=e_{m}$).

(3) $$\begin{array}{c} \frac{d^{2}T\left(x\right)}{dx^{2}}=h\frac{p}{\lambda A}\left(T\left(x\right)-T_{amb}\right) \end{array}$$

where *h* is the heat transfer coefficient of air, λ is the thermal conductivity of the chevron beam material, *p* is the perimeter of the chevron beam cross-section, *A* is the area of the chevron beam cross-section, and $T_{amb}$ is the ambient temperature. By substituting the geometrical parameters of the chevron beam and the two different thermal conductivities of SU-8 and the metallic layers in Equation (3) we can get:

(4) $$\begin{array}{c} \frac{d^{2}T\left(x\right)}{dx^{2}}=h\frac{\left(2W+4e_{m}+2e_{s}\right)}{\lambda_{s}A_{s}+2\lambda_{m}A_{m}}\left(T\left(x\right)-T_{amb}\right) \end{array}$$

where $\lambda_{s}$, $\lambda_{m}$ are the thermal conductivities of SU-8 and the metallic layers, respectively, $e_{s}$, $e_{m}$ are the thicknesses of SU-8 and the metallic layers, respectively, *W* is the width of the chevron beam, and $A_{s}$, $A_{m}$ are the areas of the cross sections of the SU-8 layer and the metallic layers, respectively. The solution for Equation (4) gives us the temperature profile along the length of the chevron beam as follows:

(5) $$\begin{array}{c} T\left(x\right)=B\exp\left(Dx\right)+C\exp\left(-Dx\right)+T_{amb} \end{array}$$

(6) $$\begin{array}{c} D=\sqrt{\frac{h(2W+4e_{m}+2e_{s})}{\lambda_{s}A_{s}+2\lambda_{m}A_{m}}}; \end{array}$$

(7) $$\begin{array}{c} B=T_{f}-T_{amb}-\frac{T_{i}-T_{amb}+\left(T_{amb}-T_{f}\right)\exp\left(DL\right)}{\exp(-DL)-exp(DL)}; \end{array}$$

(8) $$\begin{array}{c} C=T_{f}-B-T_{amb} \end{array}$$

where $T_{i}$, $T_{f}$ are the initial and final temperatures of the chevron beam. Finally, the length variation expressed in Equation (2) can be rewritten as:

(9) $$\begin{array}{c} \Delta L=\int_{0}^{L}T\left(x\right)\alpha_{eq}\;dx=\frac{\alpha_{eq}}{D}\left(B\exp\left(DL\right)-C\exp\left(-DL\right)-\left(B-C\right)\right) \end{array}$$

(10) $$\begin{array}{c} \alpha_{eq}=\frac{A_{s}E_{s}\alpha_{s}+2A_{m}E_{m}\alpha_{m}}{A_{s}E_{s}+2A_{m}E_{m}} \end{array}$$

where $E_{s}$, $E_{m}$ are the Young’s moduli of SU-8 and the metallic layers, respectively, and $\alpha_{s}$, $\alpha_{m}$ are the thermal expansion coefficients of SU-8 and the metallic layers, respectively. Equation (9) gives a relation between the parameters of the chevron-shaped actuator and its thermally induced length variation. Therefore, the effect of the thickness of the metallic layer on the length variation can be estimated, as shown in Figure 2c. The figure includes the model calculations based on Equation (9), shown in the blue dashed line, and the simulation results using Comsol shown in the red solid line. It can be confirmed from the figure that an optimal value for the thickness of the metallic layer, which can generate the maximum length variation of the chevron-beam, can be obtained. This value was confirmed to be 200 nm and it will be used in subsequent sections. In addition, the maximum opening of the microgripper can be controlled by varying the metallic layer thickness, keeping in mind that all other parameters affecting ΔL, such as the dimensions of the chevron beams and the length of the microgripper’s fingers, are fixed. On the other hand, the temperature gradient along the chevron-beam can be obtained using Equation (5), as shown in Figure 2d. The same equation can be used to verify the effectiveness of the metallic layer by verifying the effect of adding a metallic layer on the temperature gradient. Two cases of chevron beams with and without metallic layers are shown. Here, the optimal value of 200 nm for the thickness of metallic layer is used. The figure includes the model calculations based on Equation (5) for the cases with and without metallic layers, shown in the red solid line and red dashed line, respectively, and the simulation results using Comsol for the cases with and without metallic layers, shown in the blue line and blue dashed line, respectively. In the case of no metallic layer, the temperature exponentially drops along the length of the chevron-beam, which reduces its overall length variation. On the other hand, a linear drop in temperature along the length of the chevron-beam can be confirmed after adding metallic layer, which shows the advantage of adding an optimized metallic layer to the chevron-shaped actuator.

### 2.3. Fabrication of Microgripper

**Figure 3:**
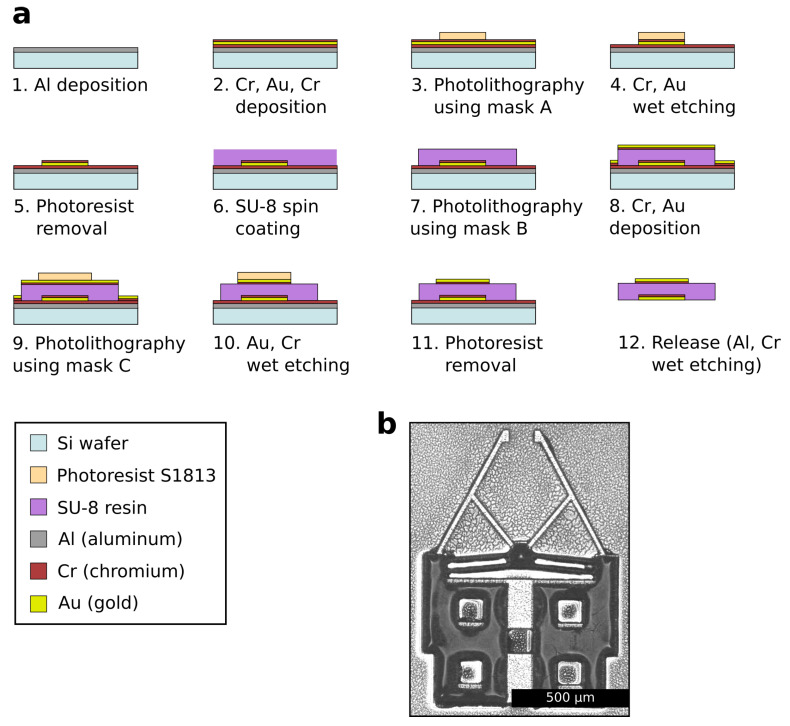
(**a**) Flowchart of the microgripper fabrication process. (1) Deposition of Al sacrificial layer. (2) Deposition of lower metallic layers. (3) First photolithography using mask A to shape the lower metallic layers. (4) Wet etching of lower metallic layers. (5) Removal of photoresist using acetone. (6) Spin coating of SU-8. (7) Second photolithography using mask B to shape the main body of the microgripper. (8) Deposition of upper metallic layers. (9) Third photolithography using mask C to shape the upper metallic layers. (10) Wet etching of upper metallic layers. (11) Removal of photoresist using acetone. (12) Wet etching of Al sacrificial layer and Cr layer to release the structures. (**b**) Image of a fabricated microgripper.

The microgripper is fabricated using conventional photolithography and metal deposition techniques. The process uses three different masks to construct the main SU-8 body and metallic layers of the microgipper. The fabrication flowchart is shown in Figure 3a, and is described as follows: (1) an 800 nm sacrificial layer of aluminum (Al) is deposited by sputtering on a silicon (Si) wafer. (2) a 350-nm thick layer of chromium (Cr), a 200-nm thick layer of gold (Au), and a 45-nm thick layer of Cr are deposited by sputtering, respectively. The chromium layers are added to enhance the adhesion between metal-metal layers and metal-polymer layers. (3) the first photolithography process using the first mask (mask A) is conducted with positive photoresist (S1813) to shape the lower metallic layers. (4) wet etching of Cr followed by wet etching of Au are conducted for 30 s and 2 min, respectively. (5) the photoresist is removed by acetone. (6) a 50 µm-thick layer of SU-8 is deposited using spin coating. Two-steps spin coating is used with speeds of 500 rpm for 30 s and 2500 rpm for 30 s, respectively. (7) the second photolithography process using the second mask (mask B) is conducted to shape the SU-8 layer. (8) a 45-nm thick layer of Cr and a 200-nm thick layer of Au are deposited by sputtering, respectively. (9) the third photolithography process using the third mask (mask C) is conducted with positive photoresist (S1813) to shape the upper metallic layers. It is worth noting that mask C is similar to mask A but with enlarged thin parts of the microgripper, such as the chevron beams, to simplify the alignment process. (10) wet etching of Au followed by wet etching of Cr are conducted for 2 min and 5 min, respectively. (11) the photoresist is removed by acetone. (12) the microgrippers are released by wet etching of Al followed by wet etching of Cr. The etching times in this step were determined visually until the removal of the Al and Cr layers are confirmed. An example of a fabricated microgripper is shown in Figure 3b.

## 3. Experiments

In this section, the behavior of the fabricated microgrippers is characterized. The optothermal response using laser actuation and the generated force of the microgrippers are evaluated. Subsequently, the functionality of the microgrippers is confirmed through a micromanipulation experiment of microbeads.

### 3.1. Microgripper Actuation and Response

**Figure 4:**
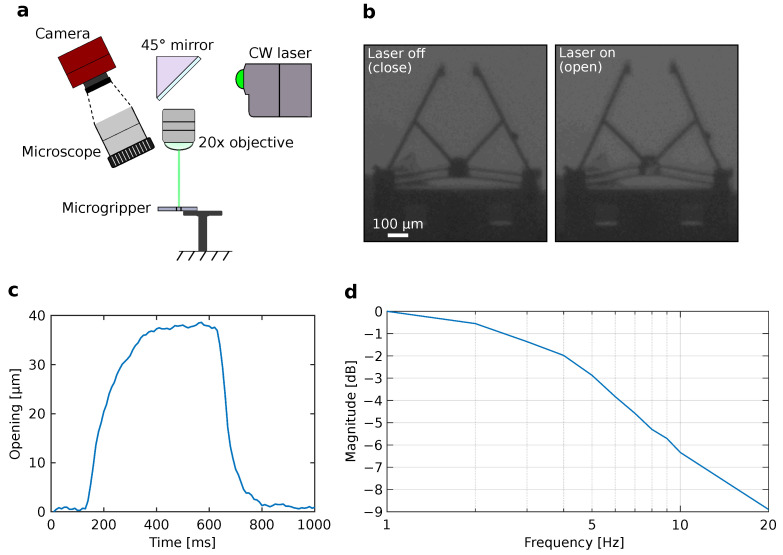
Laser actuation of the microgripper. (**a**) Experimental setup. (**b**) Experimental images showing the close and open states of the microgripper. (**c**) Step response of the microgripper. (**d**) Bode magnitude plot of the microgripper using a frequency sweep from 1 to 20 Hz.

In this section, the laser actuation of the microgripper by utilizing its optothermal response is confirmed. The experimental setup for laser actuation of the microgripper is shown in Figure 4a. The system mainly consists of a CMOS camera (Allied Vision Inc., Newburyport, MA, USA) attached to a microscope to visualize and record the actuation of the microgripper. In addition, a continuous wave (CW) laser (Oxxius Inc., Lannion, France) with a power of 53.3 mW and a wavelength of 532.1 nm was used as a laser source. The laser beam was focused using an 20× objective lens (Nikon Inc., Tokyo, Japan), where the focused laser spot size was approximately 300 µm.

Figure 4b shows the actuation of the microgripper. The microgripper is initially in a closed state without any laser heating. Upon laser heating of the chevron shuttle, the open state of the microgripper can be realized. The initial gap between the two fingers of the microgripper is approximately 100 µm (close) and then increased to 140 µm (open) by laser heating. The achieved displacement of 40 µm is approximately 1.3 times higher compared to similar designs without metallic layers deposition [17]. Figure 4c shows the step response of the microgripper. From the response, a comparatively fast response time, i.e., the time to reach 50% of the steady-state opening value of the microgripper, of approximately 60 ms is confirmed, which is 23 times faster compared to similar designs without metallic layers deposition [17]. Moreover, to confirm the frequency response of the microgripper, the change in magnitude of the opening of the microgripper in response to a frequency sweep is confirmed. Figure 4d shows the bode magnitude plot of the microgripper response. The frequency sweep is achieved using a square wave supplied to a tip/tilt mirror (Physics Instruments Inc., Karlsruhe, Germany) to steer the laser beam to and away from the chevron shuttle of the microgripper with frequencies ranging from 1 to 20 Hz. As a result, the opening of the microgripper varied according to the applied frequency. From the frequency response, a cut-off frequency, i.e., the frequency where the magnitude reaches −3 dB value, is confirmed to be approximately 5.5 Hz. This frequency modulated response can be used to apply a periodic force to grabbed cells or microobjects in future work. These results clearly show the advantage of utilizing metallic layer deposition to enhance optothermal actuation.

### 3.2. Generated Force

**Figure 5:**
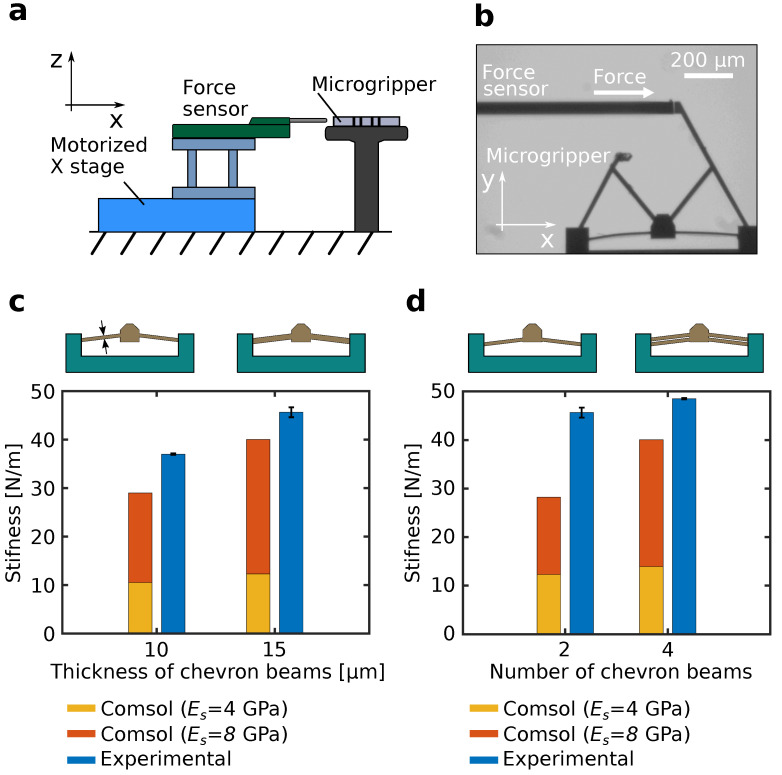
Force measurement of the microgripper. (**a**) Experimental setup. (**b**) Experimental image showing the allignment between the force sensor and a finger of the microgripper. (**c**) Effect of the width of the chevron beam on the stiffness. (**d**) Effect of the number of chevron beams on the stiffness. Blue bars show the experimental results. Orange bars and brown bars show the simulation results using SU-8 Young’s moduli of 4 GPa and 8 GPa, respectively.

For micromanipulation applications, the gripping force generated by the microgripper should be suitable to firmly hold microobjects. Therefore, the force generated by the fingers of the microgripper is investigated. However, measuring the exact force generated by the two fingers connected to the chevron-shaped actuator proves to be challenging, where a force sensing device should be placed between the fingers. Therefore, the stiffness of only one arm connected to the chevron-shaped actuator is confirmed, which gives a good estimate of the order of magnitude of the generated force. In fact, there are a number of design parameters that affects the generated force of the microgripper [17]. In this work, we investigate the effect of two parameters, namely the width of the chevron beams (*W*) and the number of chevron beams (*n*). The measurement setup is shown in Figure 5a. A force sensor (Femtotools Inc., Buchs, Switzerland) with a resolution of 0.05 µN and a range of 1000 µN is attached to a motorized x stage (Physik Instrumente Inc., Karlsruhe, Germany) with a positioning accuracy of 0.05 µm and a range of 25 mm. The microgripper is attached to a stationary holder to have the same vertical position (*z*-axis) as the force sensor. The alignment between the probe of the force sensor and the finger of the microgripper is confirmed visually as shown in Figure 5b. Subsequently, the motorized x stage is moved for 20 µm to allow the probe to push the finger of the microgripper. A data acquisition routine is initiated simultaneously to record the position of the x stage and the force sensor data. Consequently, a linear relation between the force and the stage displacement can be obtained using linear regression, where the stiffness is confirmed as the slope of the plotted curve (please refer to Figure S1 in supplementary materials). It is worth noting that the force is applied along the *x*-axis; thus only one component of the force is present. Indeed, each finger of the microgripper has two degrees of freedom (*x*-axis and *y*-axis) and it is more accurate to describe the stiffness in a matrix form to account for the coupling stiffnesses. However, this increases the complexity of the force measurement experiment and the analysis. Therefore, as the aim is to get an estimate of the order of magnitude of the force applied by the microgripper to confirm its ability to manipulate microobjects, a simple unidirectional force estimation is sufficient. A more rigorous force measurement will be planned in future work.

Figure 5c,d shows the experimental results, shown in blue bars, and simulation results using Comsol, shown in brow bars, of the stiffness of one microgripper finger. For the simulation, the commonly used value of 4 GPa for the Young’s modulus of SU-8 is used [26]. The experimental measurements are repeated four times on four different microgrippers for each plotted bar, where the error bars indicate the mean value and the standard error of the measurements. Figure 5c shows the effect of the chevron beam width on the stiffness. Two width values of 10 µm and 15 µm are investigated, where the number of chevron beams is fixed to n=2. it can be seen from the results that the width of the chevron beam had a noticeable effect on the stiffness, where an increase in stiffness of approximately 23% can be confirmed. Figure 5d shows the effect of the number of chevron beams on the stiffness. Two cases with chevron-shaped actuators having two, and four beams, respectively, where investigated, where the width of the chevron beams are fixed to W=15 µm. In this case, an increase of approximately 6% in the stiffness when doubling the number of chevron beams can be confirmed. It can be concluded from the figures that the impact on the stiffness is higher when increasing the width of the chevron beams compared to increasing the number of chevron beams. Finally, it can be noticed that the experimental results and the simulation results differ significantly. This can be due to the difference in the Young’s modulus value between the simulation and experiments, since the physical parameters of SU-8 can change according to the fabrication process. To confirm this effect, the Young’s modulus was increased in the simulation from 4 GPa to 8 GPa and the resulting stiffnesses are plotted in Figure 5c,d shown in orange columns. It is found that by increasing the Young’s modulus, stiffness values that are much closer to the experimental results are obtained. The residual difference between the experimental and simulation results can be caused by the inaccuracies in the fabrication process, such as a drift in the width and thickness of the chevron beams, or the alignment between the SU-8 layer and the gold layer. Still, more investigation on the stiffness of SU-8 using our fabrication process will be planned in future work. Overall, the experimental results serve as a good indicator for the order of magnitude of the generated force, which is in the order of hundreds of micronewtons. This force range is similar to many commercial microgrippers and is suitable for a variety of micromanipulation applications [27].

### 3.3. Application to Micromanipulation

**Figure 6:**
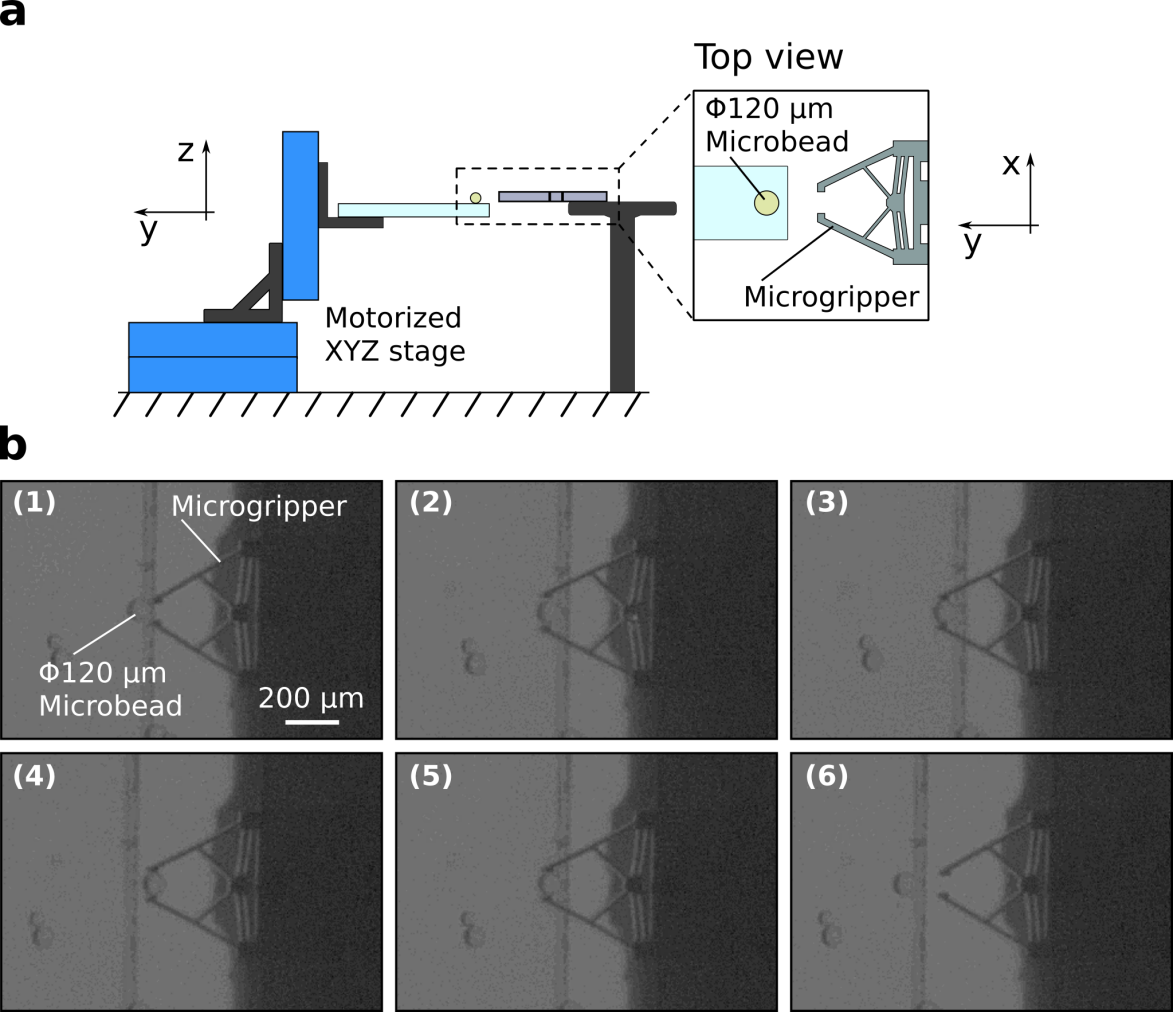
Pick-and-place of a microbead. (**a**) Experimental setup. (**b**) Snapshots of the experiment showing the successful pick-and-place of a 120-µm diameter microbead on a cover glass. See attached Video S1.

To confirm the performance of the microgripper in micromanipulation applications, a pick-and-place experiment on a 120-µm diameter microbead is performed. The microgripper incorporates two pairs of chevron beams (four beams) to enhance the gripping force. The microbead is put on a cover glass and actuated using an XYZ piezoelectric motorized stage (Physik Instrumente Inc., Karlsruhe, Germany), whereas the microgripper is attached to a stationary holder, as shown in Figure 6a. The stationary position of the microgripper allows the initiation of open/close gripping motion at any instance during the experiment. Figure 6b demonstrate the results of the pick-and-place experiment. First, the microbead is brought to the same vertical position (*z*-axis) as the microgripper, where the microgripper is in a closed state with no application of laser heating (Figure 6b-1). Next, the microgripper is switched to an open state by initiating the laser heating, and the microbead is actuated and positioned between the arms of the gripper (Figure 6b-2). Consequently, the microgripper is returned to the closed state by switching off the laser and the microbead is firmly held and actuated away from the coverglass (Figure 6b-3,b-4). Finally, the opposite maneuver is repeated to place the microbead on the cover glass again (Figure 6b-5,b-6). The successful manipulation of the microbead demonstrates the potential of the proposed microgripper to be used in micromanipulation and biomedical applications.

To get a better conclusion on the weights of objects that can be manipulated using the microgripper, the weight of the manipulated microbead is estimated analytically. As the volume and density of the microbead are known, the weight is estimated to be approximately 1 µg. In fact, many microobjects fall in this range of size and density. Specifically, biological cells are mostly equal or smaller than 120 µm in diameter with lower densities than glass. Therefore, the proposed microgripper is expected to be able to handle a large variety of microobjects including cells. The maximum weight limitation of the gripped object is mainly affected by the static friction force generated by the normal force applied on the object. This requires the estimation of the static friction coefficient, which is challenging to acquire at this small scale. Nonetheless, the generated force of the microgripper in the range of hundreds of µN is similar to many commercial microgrippers and is suitable for many micromanipulation applications.

## 4. Conclusions

In this article, we proposed a laser actuated microgripper based on a metal-coated chevron-shaped actuator for micromanipulation applications. The thermomechanical model of the metal-coated microgripper was established to identify the parameters affecting its thermal deflection. Consequently, an optimization process was conducted to find the optimal thickness of the metallic layer that achieves the largest possible deflection. Microgrippers were fabricated with SU-8 resin and gold coating using conventional photolithography and metal deposition techniques. The optothermal response and the generated force of the microgrippers were verified through characterization experiments. The microgrippers could realize a relatively large opening of 40 µm with a relatively fast response time of 60 ms. Finally, the functionality of the microgripper was demonstrated through a successful pick-and-place experiment of a microbead.

Since this kind of remotely actuated microgrippers is highly promising for mobile microrobotic application, the future direction of this work is to utilize the microgripper as a mobile microrobot to be deployed in lab-on-chip applications. To achieve this goal, the behavior of the optothermal actuation in liquid needs to be verified first. In addition, a remote actuation scheme, such as magnetic or acoustic actuation, to control the position of the microgripper needs to be integrated and tested. Moreover, as the fluidic flows generated by objects inside microfluids are significant and can affect the position of target microobjects, it is desirable to reduced the footprint of the microgripper by fabricating a miniaturized version. In this case, the size of the laser spot needs to be adjusted to be compatible with the miniaturized chevron-shaped actuator.

## Supplementary Materials

The following are available online at https://www.mdpi.com/article/10.3390/mi12121487/s1, Figure S1: Force measurement of force sensor against the automated stage displacement. Red line and black line show the experimental results and the fitted curve, respectively. Video S1: Micromanipulication of a 120 μm microbead.
