# Supplementary material for: Laser Actuated Microgripper Using Optimized Chevron-Shaped Actuator
Source: Micromachines (Basel). 2021 Nov 30;12(12):1487. doi: 10.3390/mi12121487 (PMC8706880; doi:10.3390/mi12121487)
Supplement: Supplementary file 1 [file micromachines-12-01487-s001.zip › micromachines-1475071-supplementary.pdf]

Supplementary Materials: Laser Actuated Microgripper using Optimized Chevron-shaped Actuator

Belal Ahmad, Hugo Chambon, Pierre Tissier, Aude Bolopion

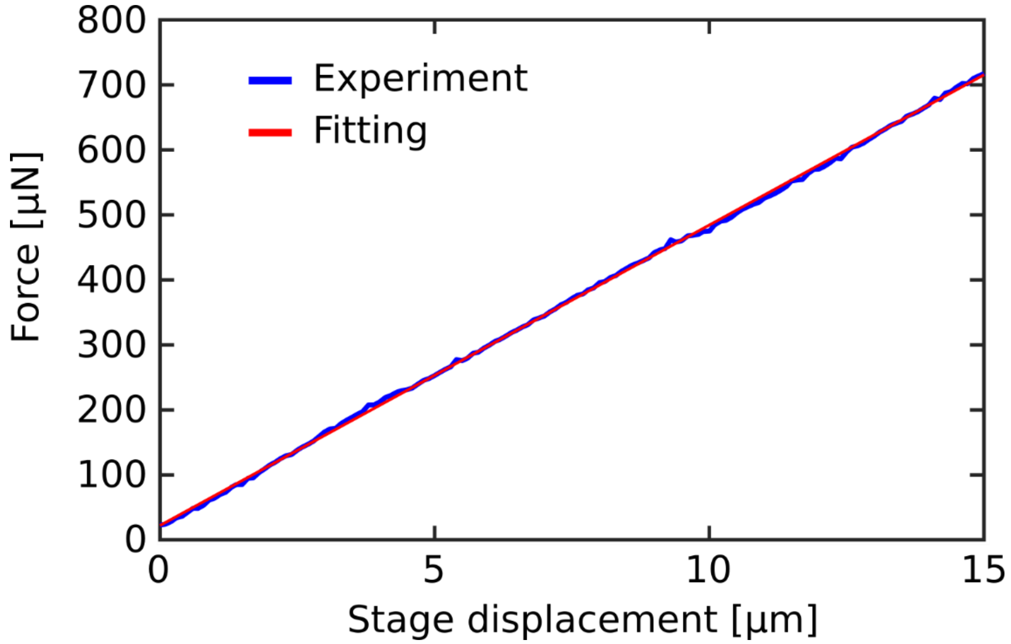

Figure S1. Force measurement of force sensor against the automated stage displacement. Red line and black line show the experimental results and the fitted curve, respectively.
